# Supplementary material for: Generative inpainting of incomplete Euclidean distance matrices of trajectories generated by a fractional Brownian motion
Source: Sci Rep. 2025 May 31;15:19145. doi: 10.1038/s41598-025-97893-5 (PMC12126505; doi:10.1038/s41598-025-97893-5)
Supplement: Supplementary file 1 — Supplementary Information. [file 41598_2025_97893_MOESM1_ESM.pdf]

Supplementary Information
Generative inpainting of incomplete Euclidean distance matrices of trajectories generated by a fractional Brownian motion

Alexander Lobashev, Dmitry Guskov, Kirill E. Polovnikov

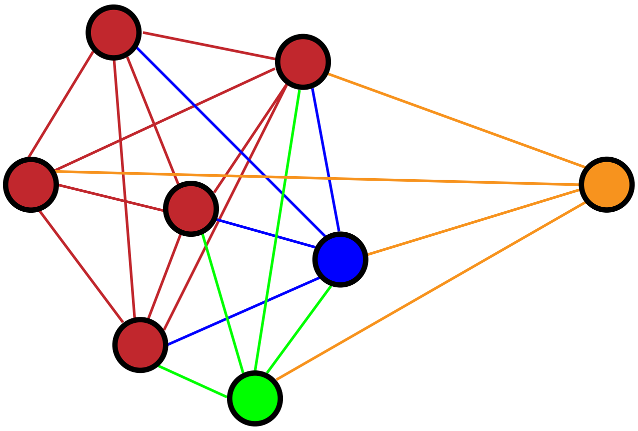

FIG. S1: A sketch of the algorithm checking for rigidity. The nodes that are added at the first step are shown in red, the blue node is added at the second, the green is added at the third, the orange one is added at the fourth step.

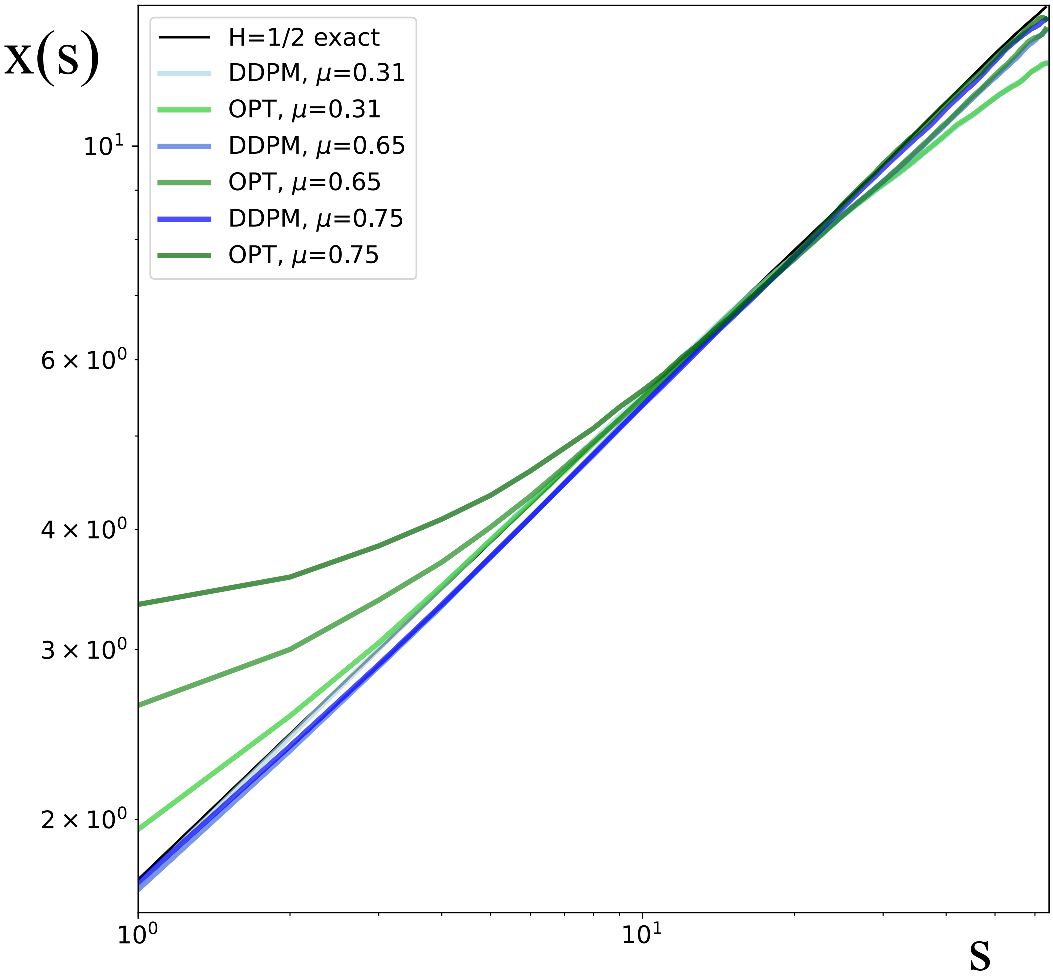

FIG. S2: Scalings of the typical spatial size $x(s)$ of the trajectory segment of the contour length s for the imputed EDM matrices using optimization (OPT) and DDPM inpainting (H=1/2). While the inpainting reproduces the theoretical behaviour at all scales s and all μ, the trajectory optimization approach tends to violate the correct scaling upon the increase of sparsity μ at small and large scales.

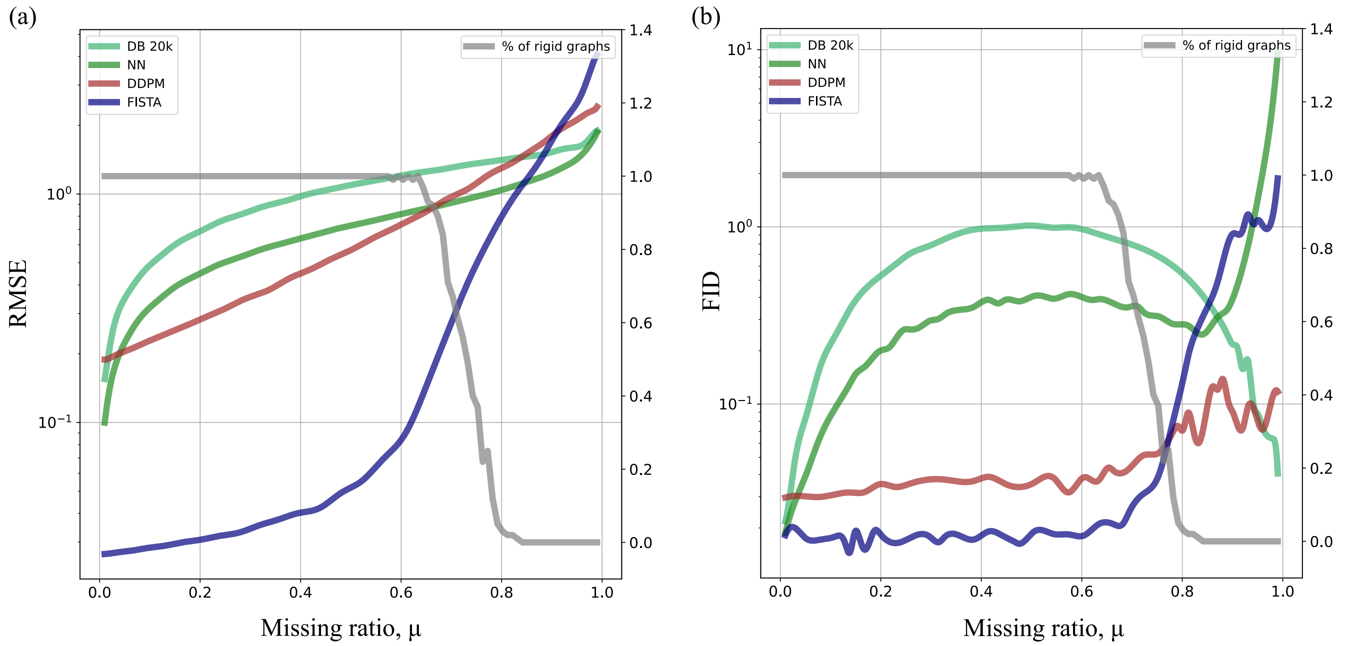

FIG. S3: RMSE (a) and FID (b) plots as a function of missing ratio μ for different data imputation methods ($H = 1/3$). The fraction of rigid graphs is shown in the second axis (grey).

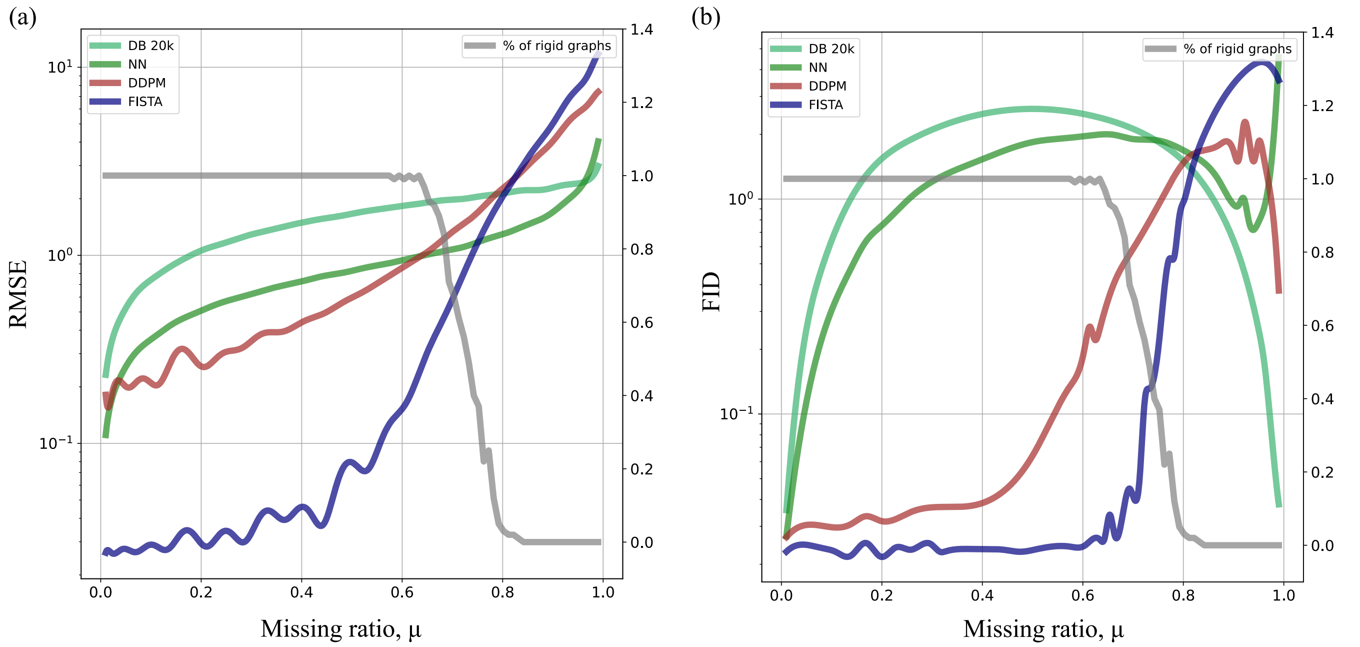

FIG. S4: RMSE (a) and FID (b) plots as a function of missing ratio μ for different data imputation methods ($H = 2/3$). The fraction of rigid graphs is shown in the second axis (grey).

**Methods**	$\frac{\sqrt{\sum_{i=1}^{5} \lambda_i^2}}{\sqrt{\sum_{i=1}^{64} \lambda_i^2}}$, $H = 1/3$	$\frac{\sqrt{\sum_{i=1}^{5} \lambda_i^2}}{\sqrt{\sum_{i=1}^{64} \lambda_i^2}}$, $H = 1/2$	$\frac{\sqrt{\sum_{i=1}^{5} \lambda_i^2}}{\sqrt{\sum_{i=1}^{64} \lambda_i^2}}$, $H = 2/3$
Database search	0.969 ± 0.011	0.982 ± 0.008	0.992 ± 0.006
Nearest Neighbour	0.982 ± 0.007	0.993 ± 0.003	0.9972 ± 0.0018
DDPM	$\mathbf{0.9977 \pm 0.0016}$	$\mathbf{0.99980 \pm 0.00018}$	$\mathbf{0.9997 \pm 0.0003}$
**Methods**	$\frac{\sqrt{\sum_{i=1}^{5} \lvert\lambda_i\rvert}}{\sqrt{\sum_{i=1}^{64} \lvert\lambda_i\rvert}}$, $H = 1/3$	$\frac{\sqrt{\sum_{i=1}^{5} \lvert\lambda_i\rvert}}{\sqrt{\sum_{i=1}^{64} \lvert\lambda_i\rvert}}$, $H = 1/2$	$\frac{\sqrt{\sum_{i=1}^{5} \lvert\lambda_i\rvert}}{\sqrt{\sum_{i=1}^{64} \lvert\lambda_i\rvert}}$, $H = 2/3$
Database search	0.73 ± 0.03	0.77 ± 0.03	0.84 ± 0.04
Nearest Neighbour	0.77 ± 0.03	0.83 ± 0.03	0.88 ± 0.03
DDPM	$\mathbf{0.91 \pm 0.03}$	$\mathbf{0.974 \pm 0.0010}$	$\mathbf{0.968 \pm 0.014}$

TABLE S1: The rank measures of the reconstructed distance matrices by different methods (database search, nearest neighbour, DDPM inpainting) at the missing ratio $\mu = 0.5$ for three values of the Hurst parameter. The measures estimate the relative contribution of the first $r = 5$ absolute values of the eigenvalues (or, squares of the eigenvalues) to the corresponding total.

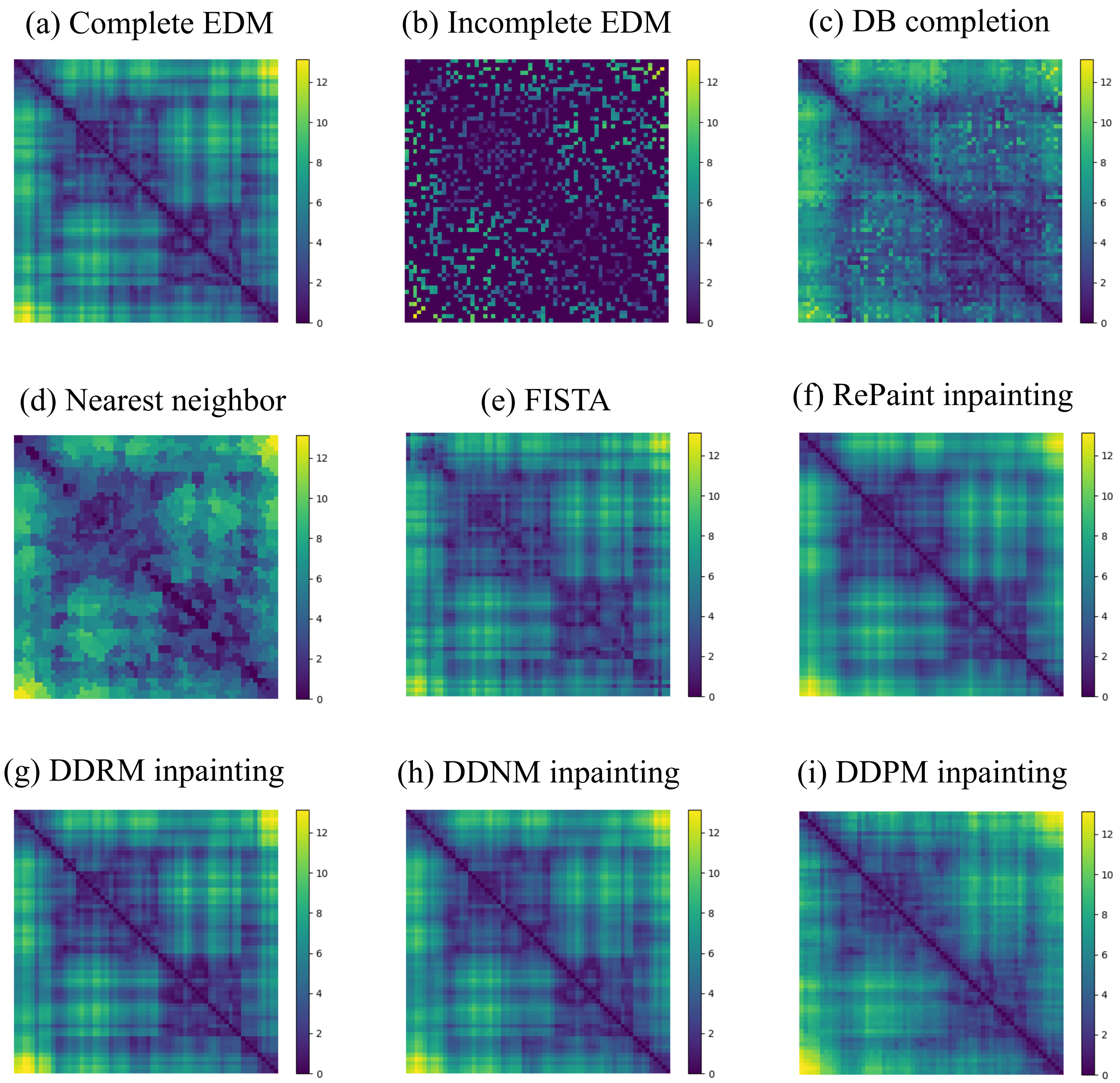

FIG. S5: Original (a), incomplete (b) matrices and completions by different methods used in the paper, as indicated. The sparsity equals to $\mu = 0.75$, for which no exact solution exists. Database completion is performed using the database size of $M = 2 * 10^4$ trajectories. The Hurst parameter is $H = 1/2$.

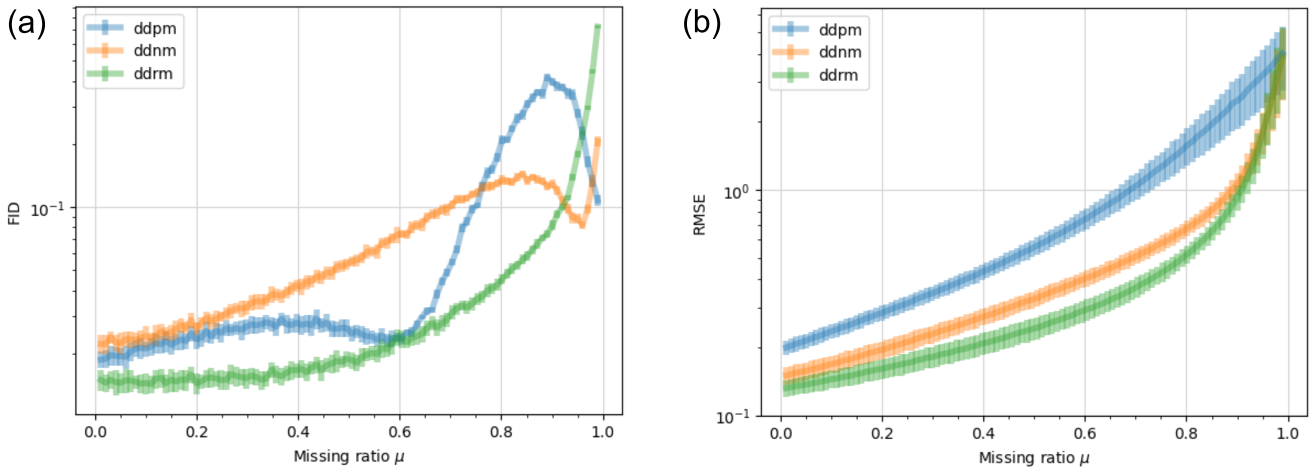

FIG. S6: (a) FID and (b) RMSE for three diffusion-based inpainting methods. The metrics are computed as functions of sparsity μ of originally incomplete EDMs of fBm trajectories. The Hurst parameter of the corresponding fBm trajectories is $H = 1/2$. The errors of RMSE are computed using a sample of 2000 inpainted distance matrices. The errors of FID for each μ are computed by randomly drawing (100 times) sub-samples with 90% of matrices and computing the values of FID for each sub-sample; then the mean and the standard deviation of these values is taken.

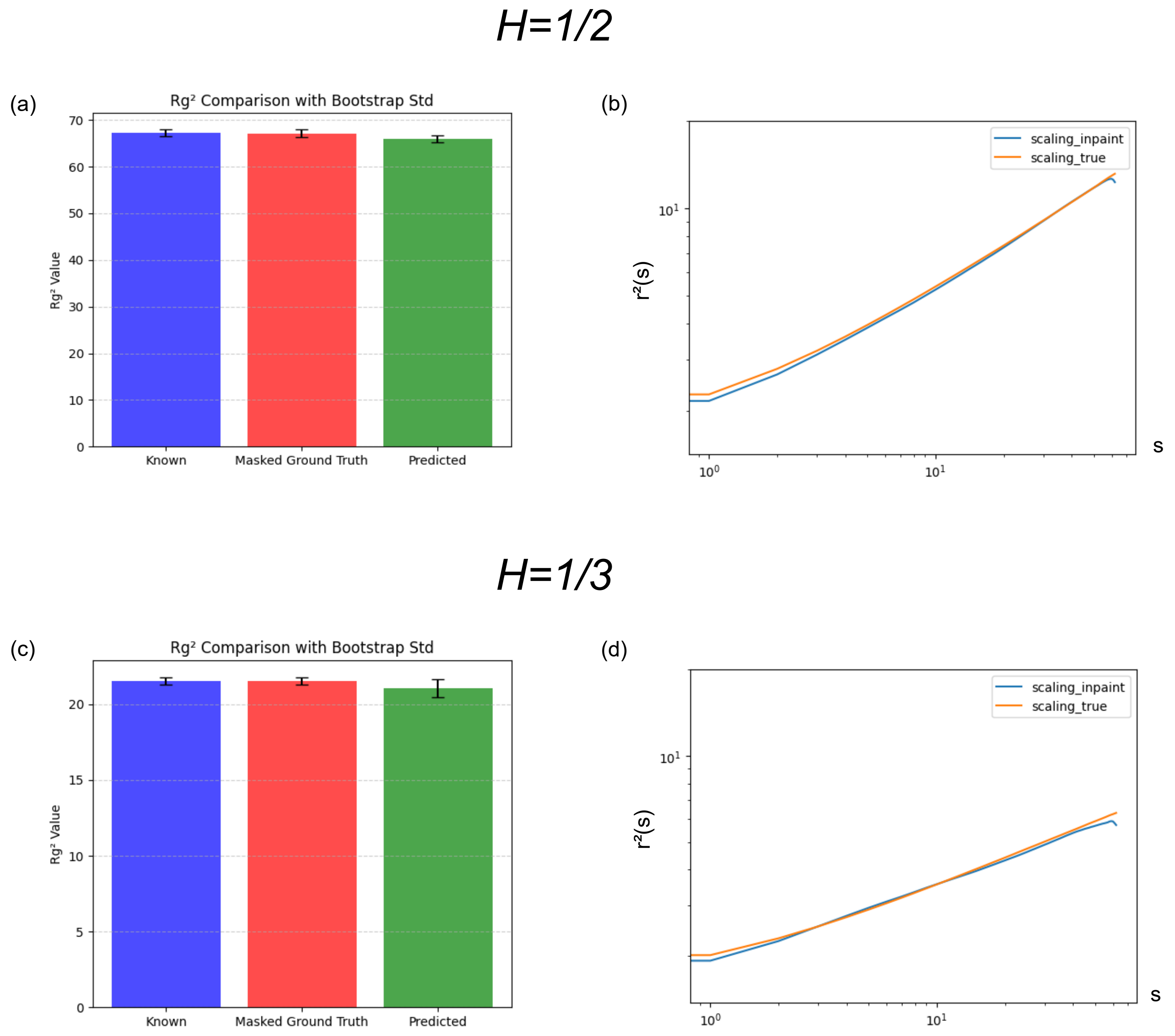

FIG. S7: Evaluation of the physical characteristics of the inpainted matrices for $H = 1/2$ and $H = 1/3$. (a,c) The average pairwise distance, represented by the squared gyration radius Rg^2, computed separately for known, masked and predicted values. (b,d) The scaling function $r^2(s)$, which represents the average distance at the diagonal s, evaluated for ground-truth and predicted values.

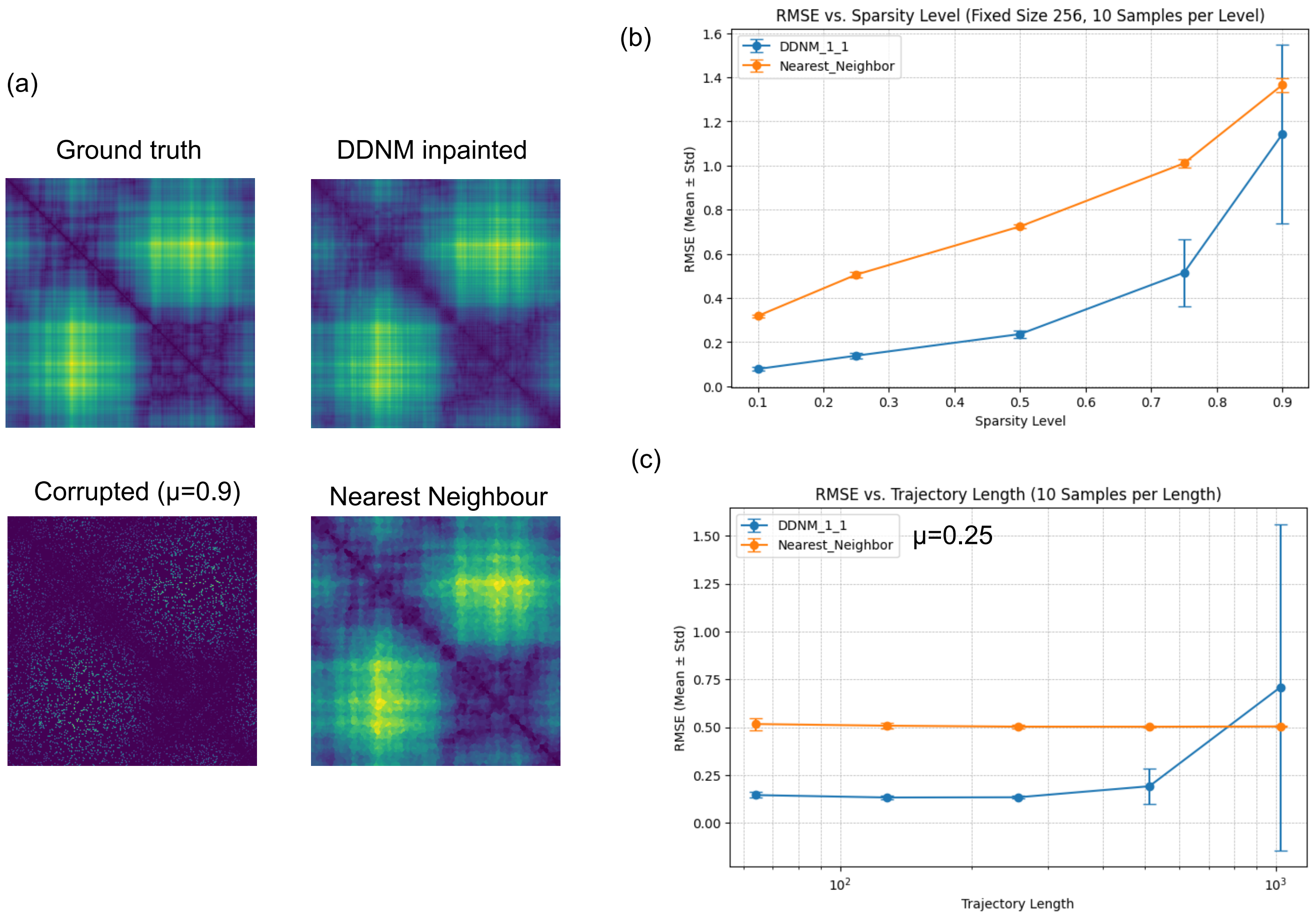

FIG. S8: Adaptation of the diffusion model trained on $N = 64$ matrices for inpainting larger matrices. (a) Inpainting results for $N = 256$ matrices at a sparsity level of $\mu = 0.9$. (b) Root Mean Square Error (RMSE) as a function of sparsity level for $N = 256$ matrices, comparing the nearest-neighbor approach and diffusion-based inpainting (DDNM). (c) RMSE as a function of matrix size at a fixed sparsity level of $\mu = 0.25$, comparing the nearest-neighbor approach and diffusion-based inpainting (DDNM).

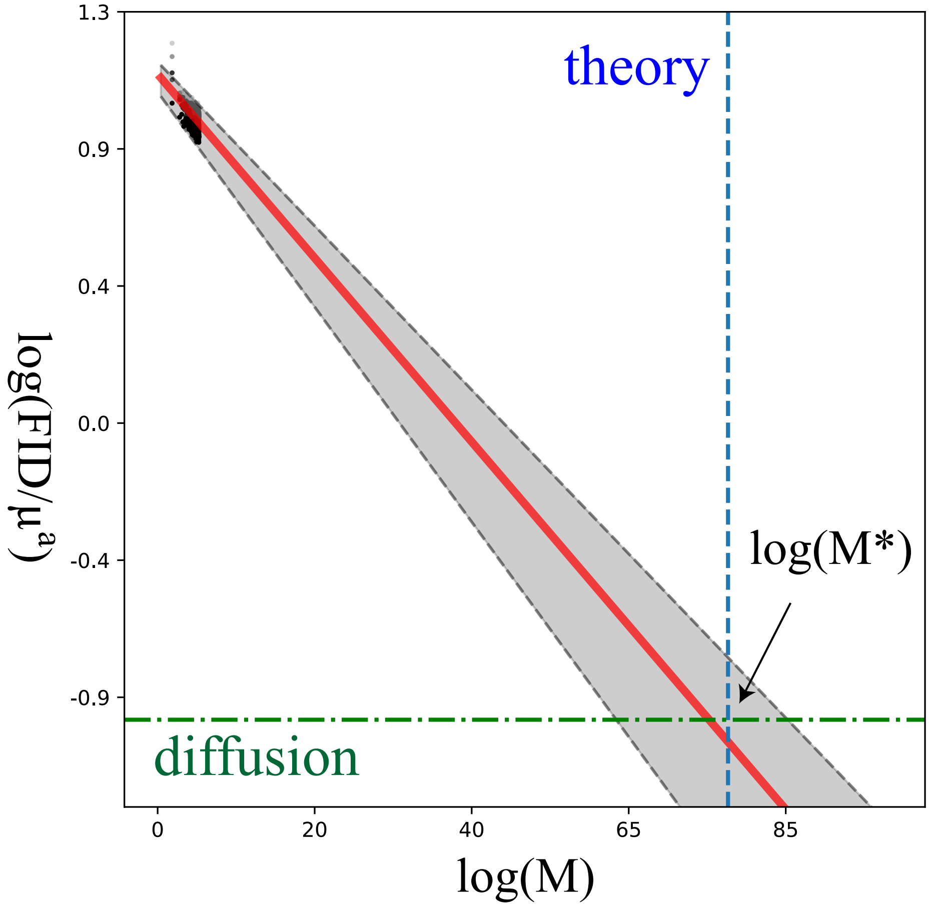

FIG. S9: Log of FID from Fig. 3(c) optimally rescaled by μ^a with $a \approx 1.4$ and further extrapolated at larger database sizes. The red line is the optimal-slope line for the whole set of points at different sparsity; the grey strip provides the lower and the upper bound for the slope. The blue dashed line is the theoretical estimation of the effective database size M^*. The green dashed line is the mean FID of the diffusion-based inpainting for the considered range of μ further scaled by $\langle\mu\rangle^a$. The arrow indicates the effective database size M^*.

Segment Index	Chromosome Index	n	Z	X	Y
24181	373	1	8482	129943	64040
24182	373	2	8441	129908	64041
24183	373	3	8394	129955	64060
24184	373	4	8430	129788	64067
24185	373	5	8553	130238	64339
24186	373	6	8444	130165	64063
24187	373	7	8298	130144	64099
24188	373	8	8326	130297	64313
24189	373	9	8252	130136	64143
24190	373	10	8424	130273	64122
24191	373	11	8335	130272	64062
24192	373	12	8396	129771	63431
24193	373	13	8450	130445	63714
24194	373	14	8586	130527	63707
24195	373	15	8625	130672	63735
24196	373	16	8454	130620	63812
24197	373	17	8201	130362	64212
24198	373	18	nan	nan	nan
24199	373	19	8463	130814	64030
24200	373	20	nan	nan	nan
24201	373	21	8775	130133	63451
24202	373	22	8341	130432	64179
24203	373	23	8208	130258	64325
24204	373	24	nan	nan	nan
24205	373	25	8312	129874	64692
24206	373	26	8113	130130	64465
24207	373	27	nan	nan	nan
24208	373	28	nan	nan	nan
24209	373	29	8107	129700	64160
24210	373	30	7949	129708	64161
24211	373	31	7288	129982	63731
24212	373	32	nan	nan	nan
24213	373	33	nan	nan	nan
24214	373	34	nan	nan	nan
24215	373	35	nan	nan	nan
24216	373	36	nan	nan	nan

TABLE S2: The 3D coordinates of 30kb segments (the center positions, in nm) on the region 28Mb-30Mb of chromosome 21 of the HCT116 cell 373 (part 1). Note that 10 nodes have been additionally dropped from the original data. (1,3,14,23,26,39,43,51,59,61)

Segment Index	Chromosome Index	n	Z	X	Y
24217	373	37	8106	130225	64104
24218	373	38	7986	129517	64128
24219	373	39	7818	129537	64071
24220	373	40	7636	129434	64172
24221	373	41	7837	129253	64606
24222	373	42	7804	129358	64108
24223	373	43	7808	129358	64126
24224	373	44	7646	129311	64315
24225	373	45	7701	129525	64315
24226	373	46	7765	129656	64470
24227	373	47	nan	nan	nan
24228	373	48	8313	129185	64169
24229	373	49	nan	nan	nan
24230	373	50	7851	129993	64434
24231	373	51	8190	129829	64263
24232	373	52	8243	130238	64363
24233	373	53	nan	nan	nan
24234	373	54	nan	nan	nan
24235	373	55	8480	130410	64230
24236	373	56	8540	130605	64418
24237	373	57	8665	130560	64501
24238	373	58	8597	130403	64485
24239	373	59	8599	130559	64144
24240	373	60	8649	130703	64029
24241	373	61	8757	130610	64206
24242	373	62	8678	130609	64309
24243	373	63	8501	130637	64242
24244	373	64	8617	130734	64197
24245	373	65	nan	nan	nan

TABLE S3: The 3D coordinates of 30kb segments (the center positions, in nm) on the region 28Mb-30Mb of chromosome 21 of the HCT116 cell 373 (part 2). Note that 10 nodes have been additionally dropped from the original data. (1,3,14,23,26,39,43,51,59,61)

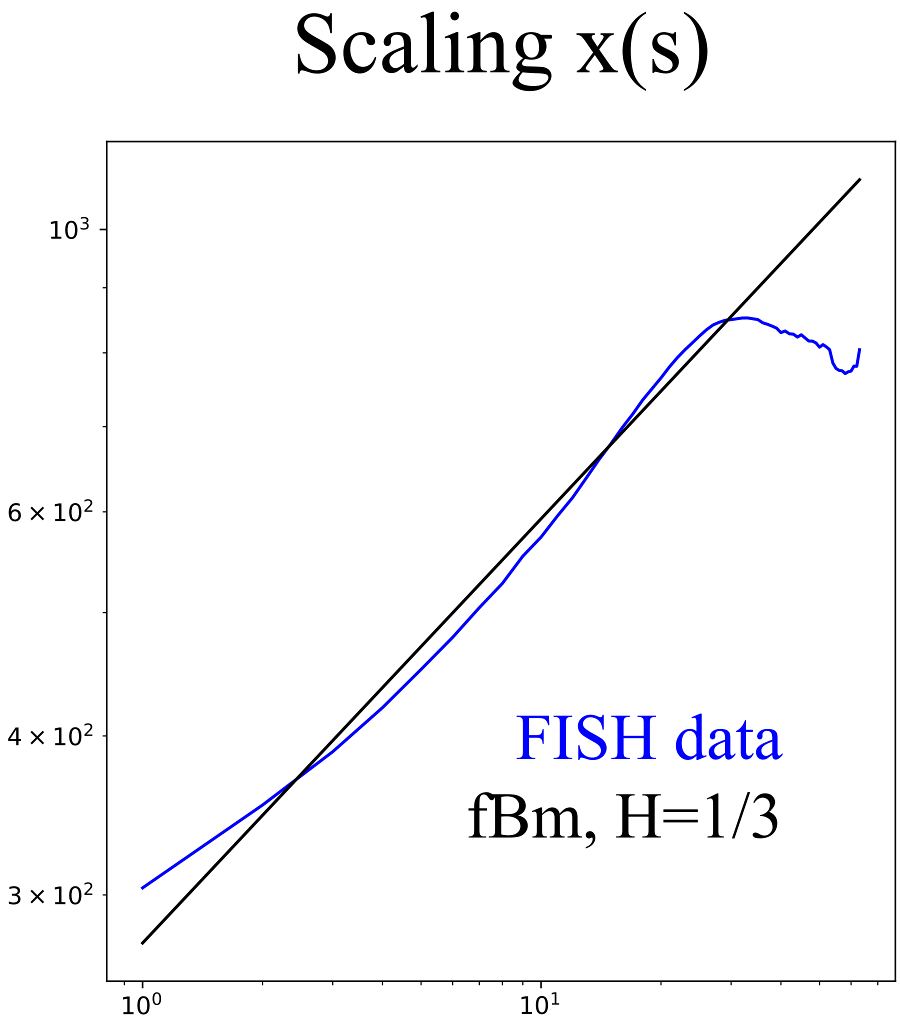

FIG. S10: Scaling of the average spatial distance $x(s)$ between two loci separated by distance s along chromosome. The spatial distance $x(s)$ is measured in nm, the chromosomal distance s is measured in 30kb bins. The blue curve is computed from the FISH data by averaging along the s-th diagonal of the matrix corresponding to the cell shown in Figure 5. The black curve corresponds to the fBm trajectory with $H = 1/3$, i.e. $\langle x_H^2(s)\rangle^{1/2} \sim s^H$, see Eq. 7.
